# Nanoscale Zeolitic Imidazolate Framework (ZIF)–8 in Cancer Theranostics: Current Challenges and Prospects

**DOI:** 10.3390/cancers14163935

**Published:** 2022-08-15

**Authors:** Hongxin Xie, Xinyu Liu, Zhengrong Huang, Liexi Xu, Rui Bai, Fajian He, Mengqin Wang, Linzhi Han, Zhirong Bao, Yuzhou Wu, Conghua Xie, Yan Gong

**Affiliations:** 1 Department of Biological Repositories, Zhongnan Hospital of Wuhan University, Wuhan 430071, China; 2 Department of Radiation and Medical Oncology, Zhongnan Hospital of Wuhan University, Wuhan 430071, China; 3 Hubei Key Laboratory of Bioinorganic Chemistry and Materia Medica, Hubei Engineering Research Center for Biomaterials and Medical Protective Materials, School of Chemistry and Chemical Engineering, Huazhong University of Science and Technology, Wuhan 430074, China; 4 Hubei Key Laboratory of Tumor Biological Behaviors, Zhongnan Hospital of Wuhan University, Wuhan 430071, China; 5 Tumor Precision Diagnosis and Treatment Technology and Translational Medicine, Hubei Engineering Research Center, Zhongnan Hospital of Wuhan University, Wuhan 430071, China

**Keywords:** nanomedicine, metal–organic framework, zeolite imidazole framework, combination cancer therapy, theranostic nanoplatform

## Abstract

**Simple Summary:**

The biomedical application of metal–organic frameworks in cancer theranostics has become a research hotspot with rapid progress. As a typical representative, ZIF–8 attracts increasing interest from researchers due to its good performance and potential. In this review, we updated recent discoveries on the ZIF–8–based nanoplatforms for cancer, discussed the problems in current research and the obstacles for clinical translation of ZIF–8, and also proposed an outlook on its future development.

**Abstract:**

Cancer severely threatens human health and has remained the leading cause of disease–related death for decades. With the rapid advancement of nanomedicine, nanoscale metal–organic frameworks are believed to be potentially applied in the treatment and biomedical imaging for various tumors. Zeolite imidazole framework (ZIF)–8 attracts increasing attention due to its high porosity, large specific surface area, and pH–responsiveness. The designs and modifications of ZIF–8 nanoparticles, as well as the strategy of drug loading, demand a multifaceted and comprehensive understanding of nanomaterial features and tumor characteristics. We searched for studies on ZIF–8–based nanoplatforms in tumor theranostics on Web of Science from 2015 to 2022, mainly focused on the research published in the past 3 years, summarized the progress of their applications in tumor imaging and treatment, and discussed the favorable aspects of ZIF–8 nanoparticles for tumor theranostics as well as the future opportunities and potential challenges. As a kind of metal–organic framework material full of potential, ZIF–8 can be expected to be combined with more therapeutic systems in the future and continue to contribute to all aspects of tumor therapy and diagnosis.

## 1. Introduction

**Figure 1:**
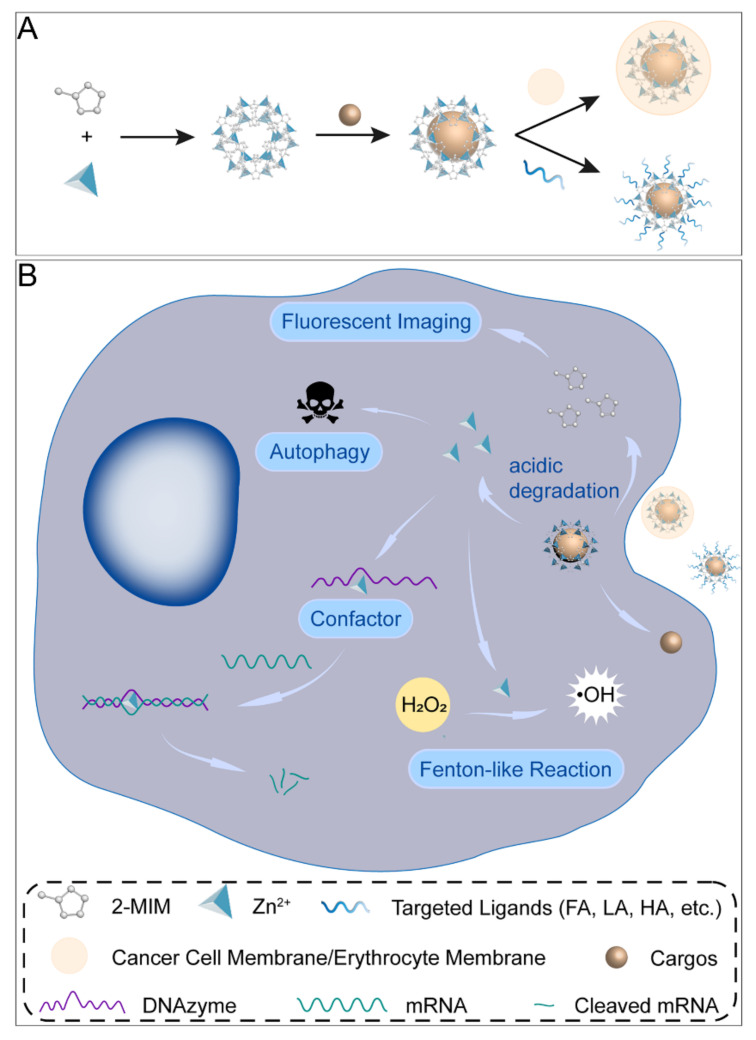
Synthesis process and decomposition of ZIF–8 nanocomposites in cancer cells. (**A**) Schematic presentation of the synthesis of ZIF–8 nanocomposites. (**B**) Illustration of ZIF–8 nanoformulations in cancer cells.

Cancer is one of the diseases that people have been dedicated to overcoming for many decades [1]. The development of emerging therapies such as targeted therapy and immunotherapy in recent years has greatly improved the survival and prognosis of cancer patients [2]. With in–depth studies of advanced nanomaterials, they are potentially applied to various tumor treatments and biomedical imaging. The zeolite imidazole framework (ZIF) is a kind of metal–organic framework (MOF) [3]. The most representative ZIF–8 is composed of zinc ions and 2–methylimidazole, which are widely studied due to their large specific surface area and thermal stability [4]. ZIF–8 is a cage–like coordination compound with regular rhombic dodecahedron crystals. The pore diameter of the sodalite cages is 11.6 Å with an aperture of 3.4 Å [5]. The synthesis methods include the room temperature solution reaction method [5], solvothermal method [6], electrodeposition–solvothermal method [7], microfluidic synthesis method [8], etc. The drug–loaded ZIF–8 is mainly constructed by the one–pot method, which can be used to in situ encapsulate drugs that are larger than the pore size of ZIF–8 and improve drug loading capacity. Research on ZIF–8–based nanoplatforms revealed the possibility of its applications in cancer therapy including immunotherapy, starvation therapy (ST), phototherapy, chemotherapy, and gene therapy (GT), as well as biomedical imaging such as magnetic resonance imaging (MRI), computed tomography (CT), and photoacoustic imaging (PAI). When applied to tumor therapy, it has the following properties: (i) high porosity and large specific surface area; (ii) relatively high biosafety; (iii) decomposition in acidic solutions, which facilitates the specific release of the drugs; (iv) generation of reactive oxygen species (ROS) by its decomposition product Zn^2+^ through Fenton–like reactions; (v) enzymatic cleavage reaction of nucleases catalyzed by Zn^2+^ as a cofactor; (vi) induction of cellular autophagy (Figure 1) [9,10]. ZIF–8 can be used as part of a nanoreactor to avoid premature drug leakage and act as a self–sacrifice template. However, ZIF–8 is poorly dispersed in water and prone to polymerization [11]. Therefore, the surface of ZIF–8 is often modified with polyethylene glycol (PEG) or hyaluronic acid (HA) [12,13]. Targeting ligand modification on the ZIF–8 surface achieved active delivery to tumor cells, and common ligands included HA, lactobionic acid (LA) [14], folic acid (FA) [15], and Arg–Gly–Asp (RGD) peptide [16]. In addition, long–time blood circulation and immune escape can also be realized by bionic mineralization on the nanodrug surface, such as coating the cancer cell or erythrocyte membrane [17,18,19]. However, existing studies have not paid enough attention to the properties of ZIF–8 itself, including the underlying mechanisms of its effects on tumor growth and the long–term toxicity in vivo. The clinical translation of the ZIF–8 drug delivery system calls for more research on ZIF–8 both in vitro and in vivo. This review summarized recent research on ZIF–8–based nanoplatforms for cancer theranostics. Moreover, their future opportunities and challenges were also discussed and highlighted.

## 2. Methods

We searched for studies on the ZIF–8–based nanoplatforms in tumor theranostics on Web of Science from 2015 to 2022, and obtained 278 articles on tumor therapy and 125 ones on tumor imaging. We mainly selected the studies published in the past 3 years, summarized the progress of its application in tumor imaging and treatment, and discussed the favorable aspects of ZIF–8 nanoparticles for tumor theranostics as well as the future opportunities and potential challenges.

## 3. Biomedical Imaging

Constructed with various metal ions or photothermal agents (PTAs), ZIF–8 nanoplatforms can be applied for CT, MRI, or PAI (Table 1). In addition to simple single–mode imaging [20,21], these rationally designed nanoplatforms enable dual–mode or even triple–mode imaging [22,23,24], thereby improving diagnostic accuracy. Moreover, targeted modification of nanocomplexes or responsive release at the tumor sites can increase their relative concentration in the tumor tissues, amplifying image contrast with normal tissues. A portion of these nanocomplexes can also carry therapeutic agents simultaneously to monitor the therapeutic effects in real time, serving as a versatile diagnostic and therapeutic platform for tumors.

### 3.1. CT

Incorporated with elements of high X–ray attenuation coefficient, ZIF–8 nanocomposites were reported to provide more accurate and clear CT images. For example, the inert element gold was commonly used to construct nanoplatforms as CT contrast agents. Zhang et al. fabricated LA–AuNR/ZIF–8 nanoparticles [14]. Due to the targeting agent LA, in vitro experiments demonstrated that HepG–2 cells have a higher nanoparticle cellular uptake rate than LA receptor–negative MCF–7 cells. After injecting LA–AuNR/ZIF–8 into the tail vein of H22–bearing mice, an in vivo CT showed strong contrast images 24 h later. Similarly, Xu et al. designed the doxorubicin (DOX)–Pt–tipped Au@ZIF–8 nanoplatform [25], which had superior photothermal and CT imaging capabilities due to the strong light absorption and X–ray attenuation of high atomic number elements such as Pt and Au.

### 3.2. MRI

Some strong paramagnetic ions such as Fe^3+^, Cu^2+^, Mn^2+^, and Gd^3+^ improve relaxation efficiency and act as T1/T2–weighted contrast agents to enhance image contrast [26,27]. Pan et al. prepared Mn–ZIF–8/5–Fu nanocomplexes for T1–weighted imaging and tumor treatment [28]. The signal intensity peaked after 12 h of intravenous injection of the nanocomplex into the tumor–bearing mice, and the enhanced signal was significantly higher than that of the control group. Notably, the clearance of Mn^2+^ from the major organs was high, with almost complete clearance of Mn^2+^ within 7 days, thus avoiding the possible long–term toxicity of the nanoparticles. Chen et al. synthesized Mn–Zn–ZIF–PEG nanoparticles for T1–weighted MRI/fluorescent imaging (FI) dual–mode imaging [29]. This study reported the fluorescent imaging ability of ZIF–8 for the first time, which might originate from 2–methylimidazole. The nanocomplexes released Mn^2+^, showing a stronger contrast in the acidic environment of the tumor than the neutral environment. The PEG modification not only increased the biosafety of the nanocomplex, but also improved its hydrophilicity and enhanced MRI effects.

### 3.3. PAI

PAI is a novel biomedical imaging method that is non–invasive and non–ionizing, with deeper penetration depth and higher resolution. Most PTAs can be used for PAI [30], where light energy is converted into heat energy by laser irradiation, causing local tissue expansion to generate pressure waves that produce photoacoustic signals. Guo et al. grew a polydopamine (PDA) shell layer on the surface of ZIF–8 wrapped with DOX and modified with Mn^2+^ and PEG [31]. Mn^2+^ is commonly regarded as an MRI contrast agent, whereas PDA is widely used for photothermal therapy (PTT) and PAI due to its good photothermal conversion rate. Both an in vivo MRI and PAI of tumor–bearing mice showed marked enhancement. Deng et al. constructed a Au@MOF nanoplatform with star–shaped gold nanoparticles as the yolk and ZIF–8 as the shell layer, equipped with DOX [32]. Under laser irradiation in the near–infrared (IR) II region (1064 nm), the gold nanoparticles exhibited excellent photothermal properties and thus could be used for IR thermal imaging and PAI. In the mouse model, the PAI signal was greatly elevated at the tumor sites of the nanoparticle–injected mice. More importantly, it has good biosafety and exhibited no significant cytotoxicity.

**Table 1:** Recent studies on ZIF–8–based nanoplatforms for biomedical imaging.

Nanocomposites	Applications	Properties	Ref.
LA–AuNR/ZIF–8	CT	high X–ray absorption coefficient (Au)	[14]
DOX–Pt–tipped Au@ZIF–8	CT	high X–ray absorption coefficient (Pt, Au)/good photothermal conversion efficiency (Pt, Au)	[25]
Mn–ZIF–8/5–Fu	MRI	enhanced relaxation (Mn)	[28]
BSA–MnO_2_/Ce6@ZIF–8	MRI	enhanced relaxation (Mn)	[33]
Fe_3_O_4_–ZIF–8	MRI	responsive T2–T1 switching MRI contrast agent (Fe_3_O_4_)	[26]
Mn_3_O_4_@PAA@ZIF–8	MRI	enhanced relaxation (Mn)	[20]
ZIF–8/DMPP	MRI/PAI	enhanced relaxation (Mn)/strong NIR absorption (PDA)	[31]
ZIF–8/DOX–PD–FA	MRI/FI	enhanced relaxation (Si–Gd NPs)/fluorescence optical imaging ability (Si–Gd NPs)	[15]
Gd/Tm–PB@ZIF–8/PDA	MRI/FI	enhanced relaxation (Gd/Tm–PB)/fluorescence optical imaging ability (Gd/Tm–PB)	[27]
Mn–Zn–ZIF–PEG	MRI/FI	enhanced relaxation (Mn)/fluorescence optical imaging ability (2–methylimidazolate)	[29]
Fe_3_O_4_@PAA/AuNCs/ZIF–8	MRI/FI/CT	enhanced relaxation (Fe_3_O_4_)/fluorescence optical imaging ability (Au)/high X–ray absorption coefficient (Au)	[23]
Au@ZIF–8	PAI	strong NIR absorption (Au)	[30]
ZCNs	PAI	strong NIR absorption (carbon nanomaterials)	[21]
PDAs–ZIF–8	PAI/IR	excellent photothermal–converted acoustic wave signals (PDA)/good photothermal conversion efficiency (PDA)	[22]
Au@MOF	PAI/IR	excellent photothermal–converted acoustic wave signals (Au)/good photothermal conversion efficiency (Au)	[32]

Abbreviations: LA, lactobionic acid; CT, computed tomography; DOX, doxorubicin; MRI, magnetic resonance imaging; BSA, bovine serum albumin; Ce6, chlorin e6; PAA, polyacrylic acid; DMPP, DOX, Mn^2+^, polydopamine, and polyethylene glycol; PAI, photoacoustic imaging; FA, folic acid; FI, fluorescence imaging; PB, Prussian blue; PDA, polydopamine; PEG, polyethylene glycol; ZCNs, ZIF–8 derived carbon nanoparticles; IR, imaging infrared.

## 4. Cancer Therapy

### 4.1. Individual Therapy

#### 4.1.1. Immunotherapy

Immunotherapy reagents include immune checkpoint inhibitors, therapeutic antibodies, and immunomodulators. Compared with conventional treatments such as radiotherapy and chemotherapy, the frequency and severity of side effects are significantly reduced. With the in–depth studies of ZIF–8, it has been more and more widely used in immunotherapy [13,34,35,36,37,38], as the carrier of immune checkpoint inhibitors, immune adjuvants, or cancer vaccines (Table 2).

**Figure 2:**
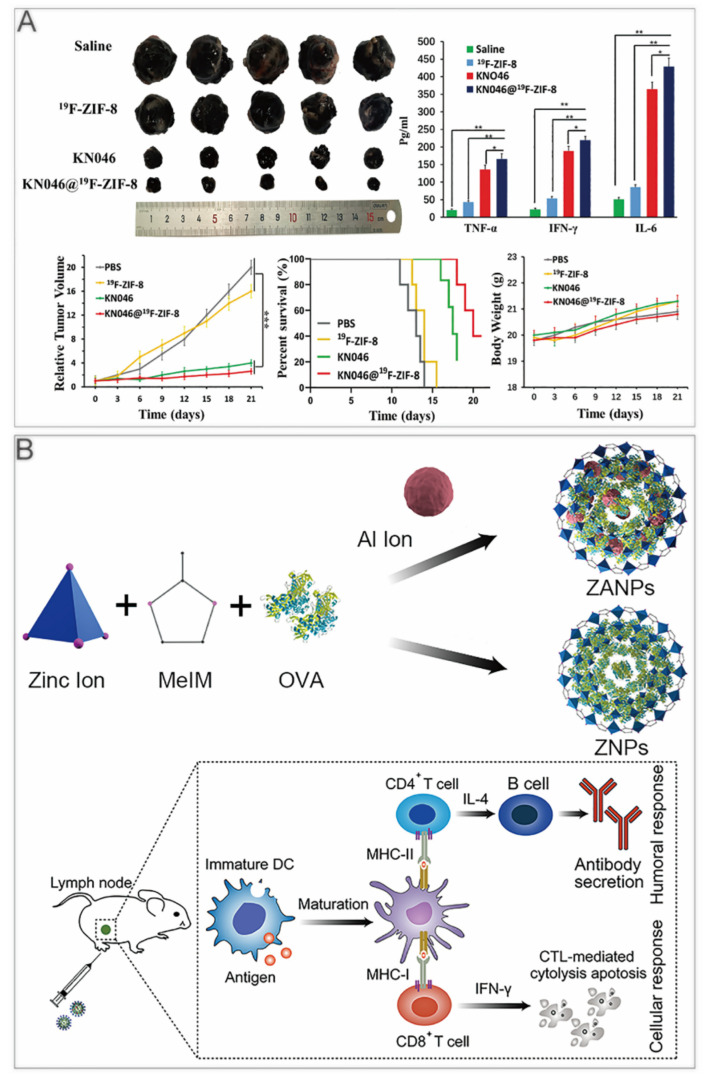
ZIF–8 nanoplatforms for immunotherapy. (**A**) KN046@^19^F–ZIF–8 inhibits tumor growth in mice. *, *p* < 0.05; **, *p* < 0.01; ***, *p* < 0.001. Reprinted with permission from ref [42]. Copyright 2021. Jiang et al. *Advanced Science* published by Wiley–VCH GmbH. (**B**) ZANPs for vaccine delivery induce antitumor immune response in immunized mice. Reprinted with permission from ref [34]. Copyright 2019 Elsevier B.V.

Immune checkpoint inhibitors are widely applied in tumor immunotherapy. They have the advantages of being long–lasting and having low toxicity [39,40]. To realize continuous drug release and effective delivery, Alsaiari et al. synthesized high–loading NV–ZIF nanoparticles to achieve a slow and continuous release of nivolumab (NV) [41]. Compared with the naked NV, the sustained releasing of NV–ZIF could activate T cells with higher efficiency. Jiang et al. used fluorine–doped ZIF–8 to coat KN046, a recombinant humanized PD–L1/CTLA–4 bispecific single–domain antibody–Fc fusion protein that can block both PD–L1 and CTLA–4 [42]. KN046@^19^F–ZIF–8 degraded in the weakly acidic tumor microenvironment to release KN046 antibodies. When the ZIF–8 structure collapsed, the ^19^F–MRI signal could be used as a probe to make tumor–specific imaging. In the tumor–bearing mouse model, KN046@^19^F–ZIF–8 significantly inhibited tumor growth (Figure 2A).

In addition to encapsulating immune checkpoint inhibitors, Zhang et al. loaded Toll–like receptor 9 agonist cytosine–phosphate–guanine (CpG) oligodeoxynucleotides (ODNs) on ZIF–8 to synthesize ZIF–8/CpG ODNs complex, which could activate innate immunity and promote cytokine secretion [43]. Due to the electrostatic repulsion between negatively charged CpG ODNs and electronegative cell membrane, the uptake rate of CpG ODNs was very low. The complex with ZIF–8 can increase the uptake rate of CpG ODNs and realize the pH–responsive release in endolysosome.

In recent years, nanocarriers have also been developed for cancer vaccines, which use tumor cell–associated antigens to awaken the body’s immune system to achieve preventive effects. Zhong et al. first reported an aluminium integrated nanoscale MOF with antigen ovalbumin (OVA) packaged in ZIF–8 (ZANPs) (Figure 2B). The researchers coated CpG on the surface of ZANPs to obtain CpG/ZANPs [34]. After the injection into the footpad of mice, in vivo near–IR fluorescence imaging showed that the time ZANPs retained in lymph nodes was greatly longer than OVA, which was at least 24 h. In vaccinated mice, the percentage of interferon–γ+ CD8+ T cells of the CpG/ZANPs group was much higher than that of other groups. Moreover, the researchers implanted EG7–OVA cells into C57BL/6 mice, then vaccinated them for 3 times every 4 days. The tumor volume and survival curves showed that CpG/ZANPs successfully slowed tumor progression.

#### 4.1.2. ST

**Figure 3:**
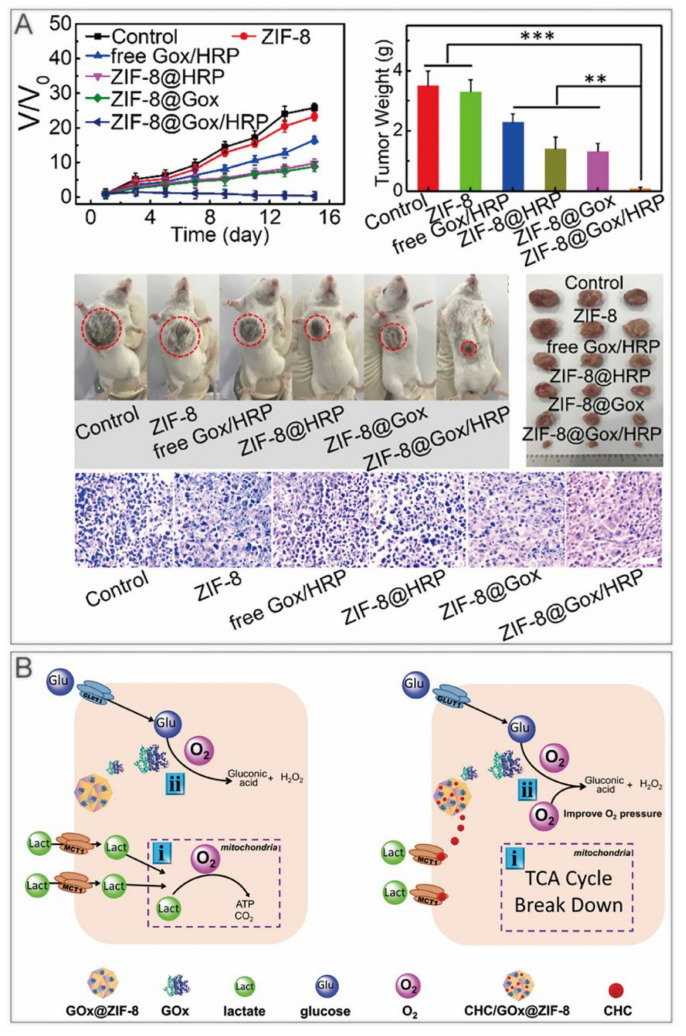
ZIF–8 nanoplatforms for individual ST. (**A**) ZIF–8@Gox/HRP inhibits tumor growth in mice. **, *p* < 0.01; *** *p* < 0.001. Reprinted with permission from ref [47]. Copyright 2019 American Chemical Society. (**B**) CHC/GOx@ZIF–8 doubly blocks the nutrient supply to cancer cell. Reprinted with permission from ref [48]. Copyright 2021 Yu et al. *Advanced Science* published by Wiley–VCH GmbH.

ST kills cancer cells by limiting their growth conditions. For example, glucose oxidase (GOx) is commonly used to deplete glucose. However, GOx is inactivated easily with a short half–life in vivo, and the consumption of glucose from normal tissues results in high levels of oxidative stress, leading to a series of side effects [44]. The device of GOx coated with ZIF–8 overcame these defects and realized the pH–responsive release of GOx in the tumor [45,46]. Some other substances comprising nano enzymes can be harbored on ZIF–8 to refine and enhance ST (Table 2). Bai et al. used the one–pot method to encapsulate horseradish peroxidase (HRP) and GOx into ZIF–8 [47]. After a week of storage, no significant aggregation appeared, demonstrating a good dispersion stability. H_2_O_2_ produced by GOx generates hydroxyl radicals in the presence of HRP, thus killing tumor cells (Figure 3A). Yu et al. fabricated the α–cyano–4–hydroxycinnamate (CHC)/GOx@ZIF–8 nanocomposite with GOx and MCT1 inhibitor CHC [48]. CHC inhibited lactate inward flow, which not only blocked one of the energy supply sources for cancer cells, but also reduced lactate metabolism to alleviate hypoxia at the tumor sites (Figure 3B).

#### 4.1.3. Photo Therapy

Tumor photo therapy, containing photodynamic therapy (PDT) and PTT [49,50,51,52,53], has received widespread attention for its advantages of non–invasiveness and low toxicity. Traditional photosensitizers or PTAs have low stability, tend to aggregate, and are difficultly internalized by cells. Hence, nanosystems constructed with photosensitizers or PTAs came into being (Table 2). ZIF–8, with its high porosity, pH–responsive release, and good biosafety, can be used to assist in the manufacture of nanosystems [54,55,56,57,58,59,60,61].

**Figure 4:**
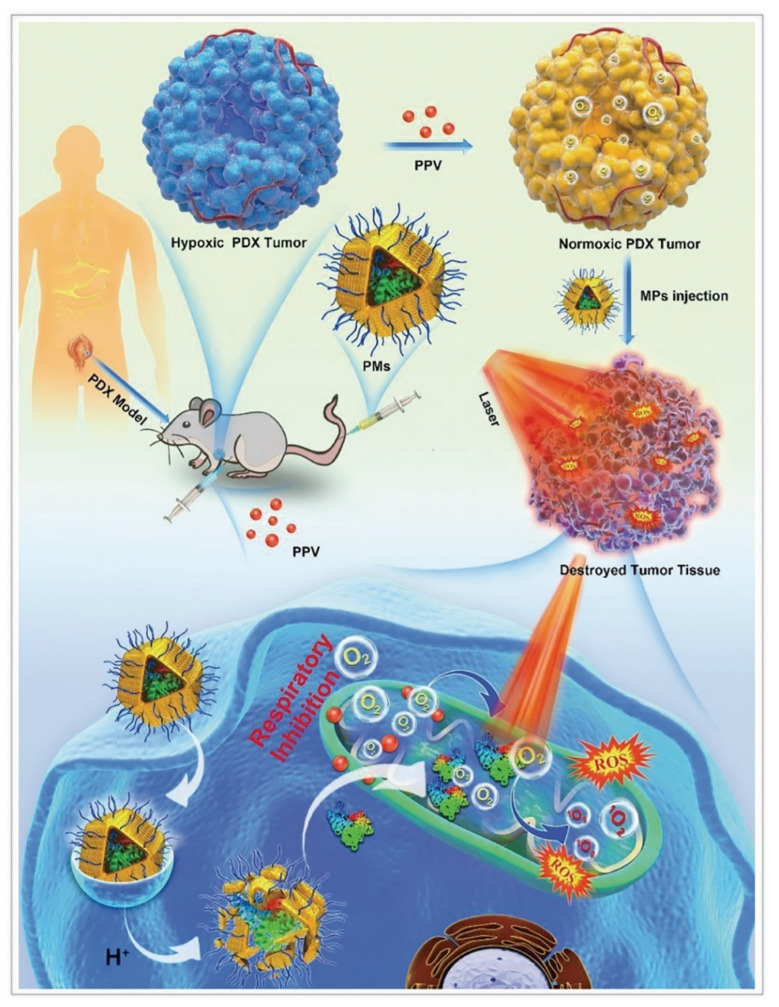
ZIF–8 nanoplatforms for individual photo therapy. Antitumor mechanism of MPEG2000–ZIF/phycocyanin composites (PMs) in the patient–derived xenograft models. Reprinted with permission from ref [66]. Copyright 2020 Wiley–VCH GmbH.

For PDT, ZIF–8 could reduce the photobleaching and dark toxicity of photosensitizers as vehicles [62]. Fu et al. synthesized ZIF–8@chlorin e6 (Ce6)–HA with an average size of 150 nm, which increased accumulation of the photosensitizer Ce6 in the tumor [63]. Xu et al. constructed zinc(II) phthalocyanine (ZnPc)@ZIF–8 with photosensitizer ZnPc by co–precipitation method [64]. The researchers optimized the amount of ZnPc to avoid the aggregation of ZnPc dispersed in ZIF–8 micropores. Although a single loading of photosensitizer can effectively avoid the hydrophobic photosensitizer aggregating and increase the uptake of photosensitizer by cells, it cannot overcome the hypoxia at the tumor sites, thus decreasing the efficiency of PDT. Ma et al. embedded AuNPs on the surface of ZIF–8 as catalase and wrapped Ce6 [65]. Sun et al. designed bovine serum albumin (BSA)–MnO_2_/Ce6@ZIF–8, in which bovine serum albumin had catalase activity [33]. In addition to Ce6, phycocyanin (PC) is a high quantum yield photosensitizer equipped with good biocompatible and light–absorbing properties, which could be extracted from Spirulina. However, as a protein photosensitizer, it is susceptible to enzymatic degradation, and its uptake by tumor cells is hindered by the identically negative charge on cell membranes. Consequently, Chen et al. fabricated MPEG2000–ZIF/PC composites by co–precipitation method, which anchored MPEG2000–COOH on the surface of the nanocomplex through the coordination between the −COOH group and zinc ions [66]. This nanocomplex protected the photosensitizer with ZIF–8, and enabled its site–specific release in the tumor, while mitochondrial complex I inhibitor papaverine was to reduce intratumor oxygen consumption and adenosine triphosphate production, thus improving PDT efficiency (Figure 4). Cai et al. developed UCNPs/MB@ZIF–8@catalase (UCNPs = upconversion nanoparticles; MB = methylene blue) nanocomposites, and the layer of catalase catalyzed endogenous H_2_O_2_ to alleviate local tumor hypoxia [67]. This nanocomposite not only encapsulated the photosensitizer well, but also promoted the adsorption of oxygen molecules on its surface.

For PTT, ZIF–8 could address the problems of poor solubility, stability, and rapid degradation of organic dyes [53]. For example, Li et al. synthesized cyanine (Cy)@ZIF–8 nanoparticles that can be used for tumor fluorescent imaging and PTT by embedding the organic dye Cy [68]. Wang et al. proposed indocyanine green (ICG)@ZIF–8 nanocomposites with excellent near–IR imaging ability and photothermal effect under laser irradiation [69]. In addition, the expression levels of heat shock proteins (HSPs) were upgraded when the organism was exposed to high temperature, increasing the heat resistance of the body. Therefore, elevated levels of HSPs in tumor cells would have unfavorable impacts on the therapeutic effects of PTT [70]. To address this issue, Li et al. loaded the HSP90 inhibitor garcinia cambogia acid onto ZIF–8 nanoparticles with bismuth nanodots. Apoptosis of cancer cells could be realized at a low temperature of 43°C by inhibiting the expression of HSP90 [71].

#### 4.1.4. Chemotherapy

**Figure 5:**
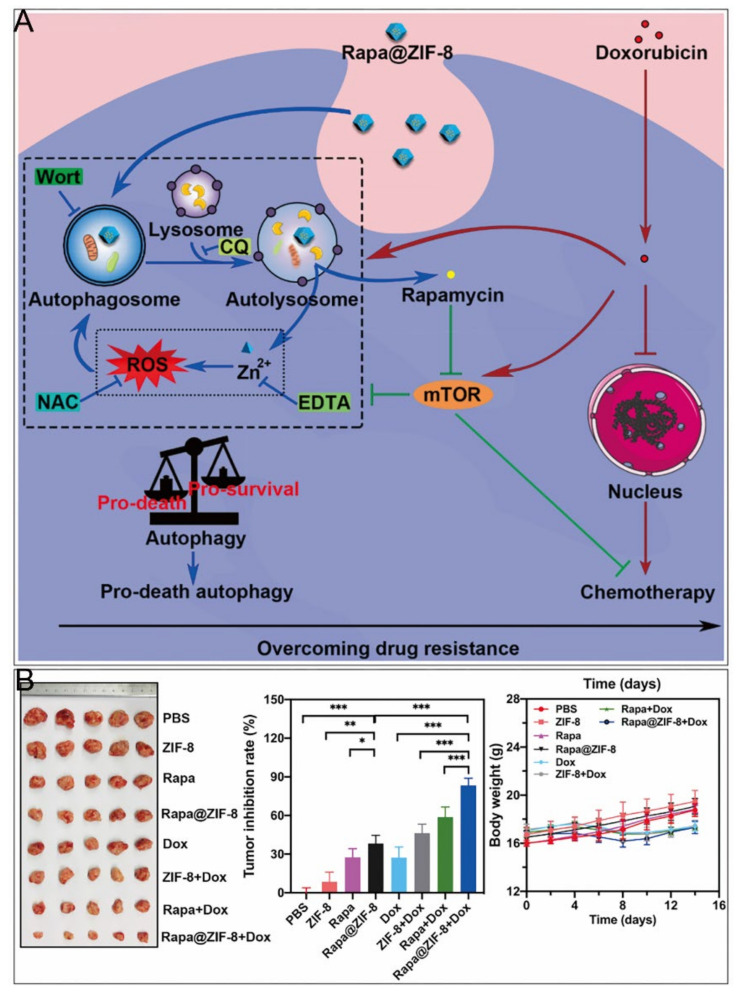
ZIF–8 nanoplatforms for individual chemotherapy. (**A**) Mechanism of overcoming drug resistance by RAPA@ZIF–8. (**B**) Antitumor effect in vivo. *, *p* < 0.05; **, *p* < 0.01; ***, *p* < 0.001. Reprinted with permission from ref [9]. Copyright 2020 Elsevier Ltd.

Delivery of chemotherapeutic drugs by ZIF–8 for specific release in tumor tissues is one of the most widely used therapeutic approaches in cancer research (Table 2) [72,73,74,75,76,77,78,79,80,81,82]. Interestingly, as many other nanomaterials have been demonstrated to elicit pro–death autophagy [83,84], Xu et al. found that ZIF–8 induced PI3K–regulated death–promoting autophagy [9]. After 24 h incubation, both autophagy inhibitors restored the diminished cell viability caused by ZIF–8. The autophagic process promoted the degradation of ZIF–8, producing cytotoxic Zn^2+^ and ROS, and the released Zn^2+^ enhanced autophagy in turn. In contrast, mTOR activation played important roles in cell survival [85,86], which were responsible for the development of resistance to many chemotherapeutic drugs such as DOX [87,88]. Accordingly, a nanoparticle encapsulating the mTOR inhibitor rapamycin (RAPA) with ZIF–8 was designed, which had a chemosensitizing effect and cooperated with DOX to restore the organism’s chemosensitivity (Figure 5). Nevertheless, ZIF–8 was revealed to induce pro–survival autophagy [10], which might result from different cell lines, concentrations of ZIF–8, or culture times.

#### 4.1.5. GT

Nucleases and non–coding RNAs, including DNAzyme [89,90], ribonuclease A (RNase A) [91,92], microRNA (miRNAs) [93], and small interfering RNA (siRNAs), regulate gene expression and achieve therapeutic purposes by silencing specific genes. However, low cellular uptake and susceptibility to degradation limit their applications. ZIF–8 can serve as a suitable carrier, delivering them to cancer cells, while the released zinc ions can also act as a cofactor for the enzymatic cleavage reaction and improve the regulatory efficiency of gene expression (Table 2).

For individual GT, Jia et al. encapsulated RNase A in ZIF–8 with an average size of 425.3 nm [91], and confocal laser scanning microscopy (CLSM) images showed that ZIF–8 effectively helped RNase A uptake by cells, and 3–(4,5–dimethylthiazol–2–yl)–2,5–diphenyltetrazolium bromide (MTT) assay confirmed that RNase A@ZIF–8 maximally inhibited tumor cell proliferation compared with single ZIF–8 and RNase A. In addition, Alyami et al. reported a nanoplatform C3–ZIF, and they used ZIF–8 as a vector to deliver the CRISPR/Cas9 gene editing elements and coated cancer cell membranes on the surface. After C3–ZIF_MCF_ was incubated with MCF–7 and other cell lines, MCF–7 showed the highest uptake, and the EGFP inhibition in MCF–7 cells transfected with C3–ZIF_MCF_ was 3 times higher than that in MCF–7 cells transfected with C3–ZIF_HELA_ [94]. These results indicated that the nanoplatform coated with cancer cell membrane had higher homotypic targeting and gene editing efficiency. Compared to the safety risk of non–target gene mutations that may be caused by virus–based vectors, utilizing nanomaterials such as ZIF–8 to transport CRISPR/Cas9 systems may be a good alternative. Further studies and applications can be expected.

**Table 2:** Recent research on ZIF–8–based nanoplatforms for individual cancer therapy.

Applications	Nanocomposites	Animal Models/Cancer Cell Types	Functions
Immunotherapy	NV–ZIF_MCF_	BALB/c mice bearing 4T1 tumors/ MCF–7/HeLa cells	a higher efficacy to activate T cells/tumor–specific targeted delivery [41]
	KN046@^19^F–ZIF–8	BALB/c mice bearing B16F10 tumors/B16F10 cells	improved the immune response rate of the antibody drug [42]
	CpG/ZANPs	C57BL/6 mice bearing EG7–OVA tumors/none	induced strong antigen–specific humoral and cytotoxic T lymphocyte responses [34]
ST	ZIF–8@GOx/HRP	Kunming mice bearing U14 tumors/ HeLa cells	interrupted the glucose–dependent energy supply/produced high toxic ROS [47]
	CHC/Gox@ZIF–8	BALB/c–Nude mice bearing SiHa tumors/MCF–7 cells	dual–blocked the main energy sources (glucose and lactate) [48]
PDT	ZIF–8@Ce6–HA	BALB/c mice bearing HepG2 tumors/HepG2 cells	increased the efficiency of PDT [63]
	ZnPc@ZIF–8	None/HepG2 cells	excellent photodynamic activity [64]
	Au@ZIF–8	BALB/c mice bearing EMT–6 tumors/EMT–6 cells	alleviated tumor hypoxia/promoted the production of ^1^O_2_ [65]
	PMs	BALB/c nude mice bearing patient–derived bladder tumors/patient–derived cancer cells	reduced intratumor oxygen consumption/increased the efficiency of PDT [66]
	BSA–MnO_2_/Ce6@ZIF–8	Kunming mice bearing U14 tumors/HeLa cells	alleviated tumor hypoxia/increased the efficiency of PDT [33]
PTT	GBZ	BALB/c nude mice bearing Huh–7 tumors/Huh–7/MCF–7 cells	achieved low temperature PTT [71]
	Cy5.5&ICG@ZIF–8–Dextran	BALB/c nude mice bearing A549 tumors/A549 cells	increased the efficiency of PTT/tumor–specific targeted delivery [53]
Chemotherapy	RAPA@ZIF–8	NOD/SCID mice bearing MCF–7/ADR tumors/MCF–7 cells	adjunct chemotherapy with the switch of survival–to death–promoting autophagy [9]
	Camptothecin@ZIF–8@RGD	None/HeLa cells	targeted and enhanced cancer treatment [16]
	HA/ZIF/DQ	BALB/c nude mice bearing HepG2/ADR tumors/HepG2 cells	remodeled the tumor microenvironment and facilitated the penetration of drug into deep tumor tissue [79]
GT	RNase A@ZIF–8	None/A549 cells	exhibited an in vitro anti–proliferative effect [91]
	C3–ZIF_(cell membrane type)_	Mice bearing MCF–7 tumors/ MCF–7/HeLa cells	improved cell–type selectivity in genome editing [94]

Abbreviations: NV, nivolumab; OVA, antigen ovalbumin; CpG, cytosine–phosphate–guanine; GOx, glucose oxidase; HRP, horseradish peroxidase; ST, starvation therapy; CHC, α–cyano–4–hydroxycinnamate; Ce6, chlorin e6; HA, hyaluronic acid; PDT, photodynamic therapy; ZnPc, zinc(II) phthalocyanine; BSA, bovine serum albumin; PTT, photothermal therapy; ^1^O_2_, singlet oxygen; PMs, MPEG_2000_ –ZIF/ phycocyanin composites; GBZ, gambogic acid/Bi@ZIF–8); Cy5.5, cyanine–5.5; ICG, indocyanine green; RAPA, rapamycin; RGD, Arg–Gly–Asp; DQ, doxorubicin and quercetin; GT, gene therapy; RNase A, ribonuclease A.

### 4.2. Dual Therapy

#### 4.2.1. Immunotherapy/PTT

When tumor cells die under external stimulation, the transformation from non–immunogenicity to immunogenicity will stimulate the antitumor immune responses, which is called immunogenic cell death (ICD) [95]. ICD was reported to increase the infiltration of immune effector cells, which can be induced by radiotherapy [96], PDT [97], PTT [98], and some chemotherapeutic drugs [99]. ZIF–8 nanocomposites were recently used for immunotherapy/PTT combination by co–loading immune reagents and PTAs (Table 3).

**Figure 6:**
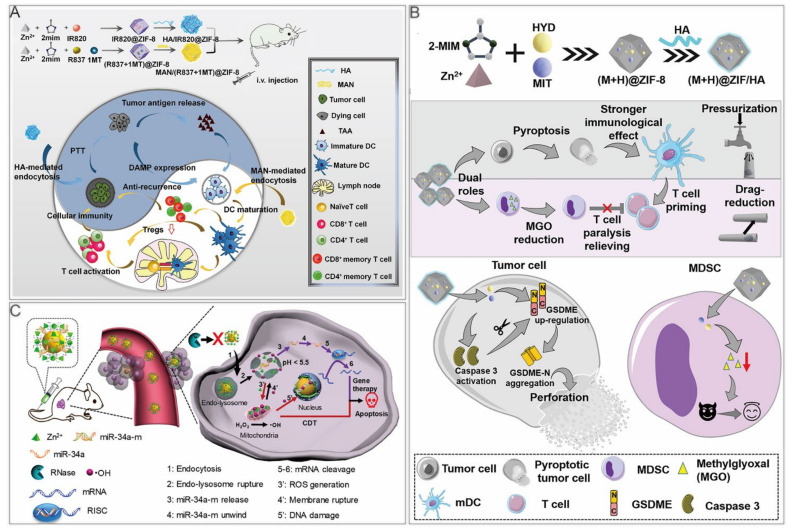
ZIF–8 nanoplatforms for dual cancer therapy. (**A**) ZIF–8 nanoplatforms for immunotherapy/PTT. Reprinted with permission from Ref [100]. Copyright 2020 Elsevier Ltd. (**B**) ZIF–8 nanoplatforms for immunotherapy/chemotherapy. Reprinted with permission from Ref [105]. Copyright 2021 American Chemical Society. (**C**) ZIF–8 nanoplatforms for GT/CDT. Reprinted with permission from Ref [93]. Copyright 2021 American Chemical Society.

Yu et al. reported a HA/ZIF–8@ICG@IMQ nanoplatform for the cold tumor treatment. A photothermal agent, ICG, combined with an immune adjuvant, imiquimod (IMQ), strengthened antitumor immunity with a long–term immune memory response [13]. Zhang et al. synthesized 2 ZIF nanoparticles, HA/IR820@ZIF–8 and mannan (MAN)/(R837+1MT)@ZIF–8 (Figure 6A) [100]. In HA/IR820@ZIF–8 nanoparticles, new indocyanine green (IR820) was a photothermal agent. Modification with HA achieved targeted delivery to tumor cells and improved cell uptake efficiency through receptor–mediated endocytosis. Under laser irradiation, tumor in situ was ablated, and the release of tumor–associated antigens (TAAs), as well as danger–associated molecular patterns, activated antitumor immunity. After treating B16F10 cells with nanoparticles under laser irradiation, the expression levels of ICD markers, such as HSP70, calreticulin, and high mobility group box protein 1 were increased, indicating that the nanoparticles promote antitumor immunity. Regarding the MAN/(R837+1MT)@ZIF–8 nanoparticles, the immune adjuvant imiquimod (R837) is the agonist of Toll–like receptor 7, whereas 1–Methyl–D–tryptophan (1MT) was an inhibitor of indoleamine 2,3–dioxygenase, which was reported to prevent the T cell proliferation via converting tryptophan to immunosuppressive kynurenine [101,102]. The nanoparticles were modified with mannan, because their receptors were highly expressed on the surface of dendritic cells. In the bone marrow–derived dendritic cells treated with nanoparticles, the expression levels of CD80 and CD86 were upregulated, and the secreted tumor necrosis factor–α and interleukin–6 were notably increased, demonstrating improved marrow–derived dendritic cell maturity. When the 2 nanoparticles were used at the same time, ICD induced by PTT promoted TAA release, thus further improving dendritic cell maturity. Moreover, the proportion of immunosuppressive Tregs was the lowest. In vitro, the simultaneous use of these 2 nanodrugs considerably inhibited the growth of primary and distant tumors, and could form immune memory. When the tumor cells were injected again, the immune memory cells in vivo resisted their attacks.

#### 4.2.2. Immunotherapy/Gas Therapy

As an emerging tumor therapy, gas therapy also prompts ICD. Common gases such as carbon monoxide (CO) can bind to haemoglobin in tumor tissues, inhibiting oxygen transportation and mitochondrial respiration. Xiao et al. designed a multifunctional CO nanogenerator CO_2_–g–C3N4–Au@ZIF–8@F127 (CCAZF), which degraded in an acidic tumor microenvironment and released CO_2_–g–C3N4–Au (CA) [103]. Under laser irradiation, CO_2_ was transformed into CO, realizing light–controlled release of CO, which reduced the toxicity of CO to normal tissues, and promoted the production of ROS, as well as the damage of mitochondria in the tumor sites, thus inducing ICD. CCAZF combined with PD–L1 antibody substantially inhibited tumor growth in the tumor–bearing mouse model (Table 3).

#### 4.2.3. Immunotherapy/Chemotherapy

Pyroptosis is programmed cell necrosis mediated by gasdermin protein, which causes strong inflammatory responses. Recent studies indicated that chemotherapeutic drugs stimulated antitumor immunity via activating caspase–3 to induce pyroptosis in Gasdermin E–expressing cancer cells [104]. Therefore, Zhou et al. co–encapsulated the chemotherapeutic drug mitoxantrone (MIT) and the DNA demethylation drug hydralazine (HYD) in ZIF–8 (Table 3). HYD upregulated Gasdermin E, while MIT induced caspase–3 activation, causing cell death. In addition, HYD inhibited methylglyoxal, a metabolic marker of myeloid–derived suppressor cells, which were involved in immune paralysis of CD8+ T cells [105]. By co–loading HYD and MIT on ZIF–8 nanoparticles, immune escape was suppressed and antitumor immune effects were enhanced (Figure 6B).

#### 4.2.4. GT/Chemo–Dynamic Therapy (CDT)

The mature miRNAs in the cytoplasm can form a miRNA–induced silencing complex together with various proteins at the 3′–UTR region of target mRNAs, blocking translation or even directly degrading mRNAs. ZIF–8 was used as a nanocarrier by Zhao et al. to deliver miRNAs into cells (Table 3). ZIF–8 protected naked miRNAs from being cleaved by ribonucleases in the blood circulation. After being taken up by cells, miR–34a–m@ZIF–8 released zinc ions and miR–34a–m in the acidic environment of lysosomes, and zinc ions generated ROS through a Fenton–like reaction, leading to the rupture of lysosomes that induced further Zn^2+^ release through positive feedback (Figure 6C) [93]. In the miR–34a–m@ZIF–8 treated group, real–time quantitative polymerase chain reaction and immunoblotting showed significant downregulation of Bcl–2 mRNA and protein levels, suggesting its superior gene silencing efficiency. In the mouse model, the efficacy of the miR–34a–m@ZIF–8 group was superior to that of the miR–34a–m plus ZIF–8 group.

#### 4.2.5. GT/Chemotherapy

To avoid the side effects of traditional chemotherapy and block tumor metastasis, Wang et al. packed human early growth response–1 targeted DNAzyme into Cu/Zn bimetallic MOF nanoparticles (Table 3); under the lysosomal acidic environment, the nanoparticles disintegrated and released Cu^2+^, Zn^2+^, and DNAzyme. Cu^2+^ was reduced to Cu^+^ with the help of sodium ascorbate, triggering the copper–catalyzed azide–alkyne cycloaddition reaction, which produced resveratrol derivatives to kill cancer cells, and Zn^2+^ was involved in the DNAzyme cleavage of human early growth response–1 (EGR–1) mRNA as a cofactor, inhibiting the proliferation and migration of cancer cells [89]. Intracellular drug synthesis guaranteed biological safety. Moreover, as shown in the relative tumor volume in the mouse model, DNAzyme@Cu/ZIF–8 was much more effective than DNAzyme@ZIF–8.

### 4.3. Triple Therapy

#### 4.3.1. Immunotherapy/PTT/ST

In addition to the combination of immunotherapy and PTT, Wang et al. designed CuCo(O)/GOx@PCNs, proving the viability of immunotherapy/PTT/ST triple therapy based on the novel nanoformulations (Table 3). First, they grew ZIF–67 on the surface of the synthesized Cu/ZIF–8, then ZIF was pyrolyzed in nitrogen, forming Cu–doped cobalt oxide and porous carbon nanocomposites (CuCo(O)@PCNs] after calcination in air. Finally, CuCo(O)/GOx@PCNs hybrid nano–enzyme was gained after GOx loading in porous carbon. When the hybrid nano–enzyme acted in the tumor sites, CuCo(O) reacted with H_2_O_2_ to produce oxygen, while GOx consumed glucose to produce H_2_O_2_ with the help of oxygen, amplifying the effects of ST. In addition, porous nanocarbon has a photothermal conversion efficiency of up to 40.04% [98]. Under laser irradiation, it has the following effects: (i) ablating the primary tumor; (ii) increasing the activity of GOx; (iii) promoting the release of TAAs and increasing the maturity of dendritic cells, thus recruiting and activating T cells. Activated cytotoxic T cells can inhibit the growth of primary and distant tumors. Compared with immunotherapy/PTT dual therapy, the introduction of GOx not only depleted the main nutrition source for tumor cells, but also utilized photothermal conversion to enhance its own glycolysis.

#### 4.3.2. ST/CDT/PDT

**Figure 7:**
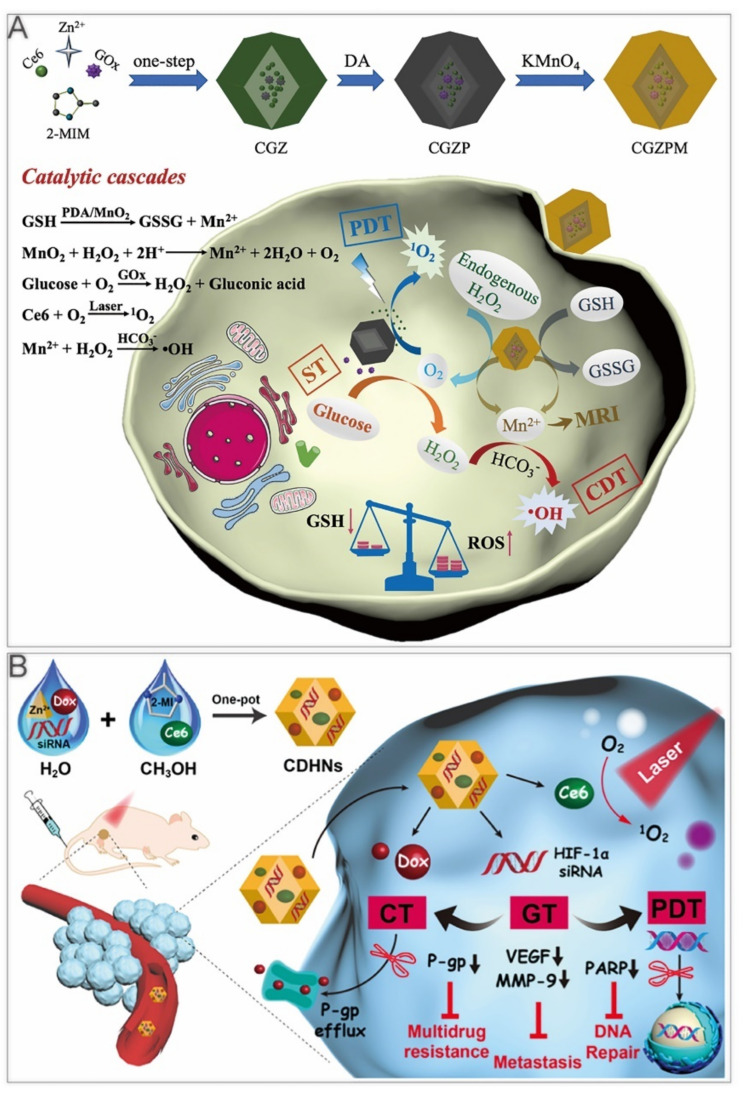
ZIF–8 nanoplatforms for triple cancer therapy. (**A**) ZIF–8 nanoplatforms for ST/CDT/PDT. Reprinted with permission from Ref [106]. Copyright 2021 Elsevier Inc. (**B**) ZIF–8 nanoplatforms for GT/PDT/Chemotherapy. Reprinted with permission from Ref [108]. Copyright 2020 American Chemical Society.

The reaction of glycolysis boosts an acidic environment in the tumor, which favors the Fenton reaction to generate free radicals. Furthermore, the warming effects caused by photothermal therapy lift the reaction rate of GOx and CDT. Since hypoxia and overproduction of glutathione (GSH) in tumor microenvironments tend to impede the efficacy of conventional PDT/CDT, Zhang et al. adopted the one–pot method to design a nanoreactor, Ce6/GOx@ZIF–8/PDA@MnO_2_ (CGZPM, Table 3). MnO_2_ reacted with H_2_O_2_ and scavenged GSH to produce Mn^2+^ and O_2_. Mn^2+^ was used as a Fenton–like reagent, while Ce6 acted as photosensitizers [106]. The O_2_ generation alleviated tumor hypoxia, enhancing PDT efficacy and expediting the glycolysis process. The PDA–covered surface improved its stability and consumed GSH together with MnO_2_ (Figure 7A). Under laser irradiation, CGZPM exhibited the strongest suppression efficacy of tumor growth in a mouse model of colorectal cancer.

#### 4.3.3. GT/PDT/Chemotherapy

Tumor hypoxia has been reported to be associated with metastasis and resistance to therapy [107]. Therefore, Wang et al. used ZIF–8 to deliver Ce6, DOX, and HIF–1α siRNA into the cells, synthesizing multifunctional Ce6–, DOX–, and HIF–1α siRNA–loaded ZIF–8 nanoparticles (CDHNs, Figure 7B). Due to HIF–1α siRNA, immunoblotting showed that the expression of vascular endothelial growth factor, matrix metallopeptidase 9, and poly(ADP–ribose) polymerase (PARP) were inhibited, suppressing the multidrug resistance, tumor metastasis, and DNA damage repair [108]. In vitro experiments have demonstrated that the GT improved the efficacy of chemotherapy and PDT, which were related to the inhibition of the P–glycoprotein (P–gp)–mediated multidrug resistance and PARP–mediated DNA repair, respectively. The CDHNs had been verified to have no obvious toxicity and prevented micrometastatic lesions in tumor–bearing mice. Light–directed drug release reduced side effects of conventional chemotherapy. Photochemotherapy sensitized by GT exerts synergistic effects through a more versatile nanosystem.

### 4.4. Quadruple Therapy

#### ST/CDT/PTT/ Immunotherapy

The combination of ST, CDT, and PTT has been reported to trigger a cascade amplification effect [109]. However, Zhang et al. designed a Fe_3_O_4_@ZIF–8/GOx@MnO_2_ (FZGM) nanocomplex and applied it to ST/CDT/PTT/immunotherapy quadruple therapy (Table 3). Due to the magnetic targeting of Fe_3_O_4_, the FZGM nanocomposites were directed to the tumor site. The ferrous ions released by Fe_3_O_4_ underwent the Fenton reaction, while Fe_3_O_4_ also generated heat under laser irradiation, not only ablating the tumor but also increasing the rate of glucose consumption of the nano–enzyme and the Fenton reaction [110]. The gluconic acid produced by the glycolysis reaction promoted the degradation of ZIF–8 in tumor cells. In addition, the synergistic effects of ST, CDT, and PDT induced ICD, encouraging the release of TAAs and converted M2 macrophages, which secreted immunosuppressive cytokines to M1 macrophages that participated in the positive immune responses. By improving the immune status of the tumor microenvironment, the efficacy of immune checkpoint inhibitors can also be strengthened. Under laser irradiation and magnetic field, FZGM markedly suppressed the primary tumor growth in the mouse model. However, the combination group containing α–PD–1 antibody and FZGM maximized inhibition of distant tumors.

**Table 3:** Recent research on ZIF–8–based nanoplatforms for combination cancer therapy.

Applications	Nanocomposites	Animal Models/Cancer Cell Types	Functions
Immunotherapy/PTT	ZIF–PQ–PDA–AUN	BALB/c mice bearing 4T1 tumors/ 4T1 cells	boosted both the innate and adaptive immune reactions [36]
	HA/ZIF–8@ICG@IMQ	BALB/c mice bearing CT26 tumors/ CT26 cells	built a long–term immune memory response to inhibit tumor rechallenge and recurrence [13]
	HA/IR820@ZIF–8 MAN/(R837+1MT) @ZIF–8	C57BL/6 mice bearing B16F10 tumors/B16F10 cells	prevented immune evasion [100]
Immunotherapy/Gas Therapy	CCAZF	BALB/c mice bearing 4T1 tumors/ 4T1 cells	regression of primary and distal tumors [103]
Immunotherapy/Chemotherapy	(M+H) @ZIF/HA	BALB/c mice bearing 4T1 tumors/ 4T1 cells	suppressed immune escape/built a long–term immune memory response against metastasis [105]
GT/CDT	miR–34a–m@ZIF–8	Kunming mice bearing MDA–MB–231 tumors/MDA–MB–231 cells	enhanced cancer cell apoptosis and suppressed tumor growth [93]
GT/Chemotherapy	DNAzyme@Cu/ZIF–8	BALB/c nude mice bearing MCF–7 tumors/MCF–7 cells	intracellularly synthesized drug molecule/cleaved the oncogene substrate [89]
Immunotherapy/PTT/ST	CuCo(O)/GOx@PCNs	Kunming mice bearing 4T1 tumors/ 4T1 cells	three–in–one functions of oxygen supply, glucose consumption, and photothermal conversion/regression of primary and distal tumors [98]
ST/CDT/PDT	CGZPM	BALB/c mice bearing 4T1 tumors/ 4T1 cells	improved the treatment outcome via self–accelerated cascade reactions [106]
GT/PDT/Chemotherapy	CDHNs	BALB/c nude mice bearing MDR/MCF–7 tumors/MCF–7 cells	damaged DNA immobilization/multidrug resistance elimination/and metastasis suppression [108]
ST/CDT/PTT/Immunotherapy	Fe_3_O_4_@ZIF–8/GOx@MnO_2_	Kunming mice bearing 4T1 tumors/ 4T1 cells	cascade amplification of the therapeutic effect/killed primary tumor and inhibited distant metastasis [110]

Abbreviations: PQ, varoglutamstat; AUN, an anti–programmed cell death–1 peptide; PDA, polydopamine; PTT, photothermal therapy; ICG, indocyanine green; IMQ, imiquimod; HA, hyaluronic acid; IR820, new indocyanine green; R837, imiquimod; 1MT, 1–Methyl–D–tryptophan; MAN, mannan; CCAZF, CO_2_–g–C3N4–Au@ZIF–8@F127; M+H, mitoxantrone and hydralazine; GOx, glucose oxidase; ST, starvation therapy; CuCo(O)@PCNs, Cu–doped cobalt oxide and porous carbon nanocomposites; CDT, chemo–dynamic therapy; GT, gene therapy; PDT, photodynamic therapy; CGZPM, chlorin e6/Gox@ZIF–8/PDA@MnO_2_; CDHNs, Ce6–, DOX–, and HIF–1α siRNA–loaded ZIF–8 nanoparticles.

## 5. Challenges

Despite the relatively high biosafety of ZIF–8, when the concentration exceeded the critical threshold, ZIF–8 caused severe DNA damage [111]. Accordingly, strict control of its concentration and further studies in vivo are required. In addition, researchers found that ZIF–8 caused cellular autophagy; nevertheless, whether it caused pro–survival autophagy or pro–death autophagy was still inconclusive, and maybe the different results resulted from different cell types or other experimental conditions. However, there is no doubt that these differences interfere with experimental results when ZIF–8 is constructed as a nanocarrier. As a result, more research is required to provide reference ideas for the preparation of future nanoplatforms. Moreover, whether the synthesis methods and preservation conditions of different ZIF–8 drug–loaded nanoplatforms affect the reproducibility and stability of studies is still to be further explored, as the safety and stability of nanoplatforms are crucial for clinical translation. Although these increasingly elaborate designs of nanomedicines have yielded good results, simultaneous loading of multiple cargoes on ZIF–8 increases the complexity of the synthesis process. Furthermore, whether the loaded multiple cargoes interact with each other and can be released in an orderly manner may require a more comprehensive and systematic assessment.

In addition, the size of ZIF–8 nanoparticles ranges from tens to hundreds of nanometers, and for particles between 10 nm and 200 nm, they can be passively targeted to tumor tissues through enhanced permeability and retention effect; however, most nanoparticles loaded with multiple proteins and chemotherapeutic drugs tend to exceed this range, and the excessive size is not conducive to uptake. For these particles, biomimetic mineralization or surface coating may be a good option. For example, cancer cell membranes, erythrocyte membranes, and platelet membranes, which have attracted much attention in recent years, can prolong the circulation time through immune evasion. Surface modification of FA, HA, and RGD peptides can recognize corresponding receptors highly expressed in tumor cell membranes, thus increasing the uptake of nanoparticles by tumor tissues. PEG and polyvinylpyrrolidone coatings can protect the physicochemical properties and functions of nanoparticles as well as counteract the clearance of reticulo–endothelial systems.

ZIF–8 drug delivery systems are still a long way from clinical translation, although in vitro and animal studies have confirmed their safety and efficacy, long–term in vivo safety evidence and preclinical studies are still lacking. More experiments on a wider range of tumor types, including patient–derived xenograft models, are necessary, and more research is needed to determine whether and to what extent differences in tumor cell types and tumor microenvironments affect the efficacy of nanoparticle therapy. In addition, it is necessary to optimize and standardize the synthesis method to increase yield and reduce costs. 

## 6. Prospects and Conclusions

ZIF–8 is regarded as a drug delivery platform with great potential due to its high porosity and low toxicity. Compared to the currently clinically approved contrast agents, such as iodine or gadolinium formulations, the ZIF–8 nanoplatform can be combined with targeting groups or other probes to achieve targeted and multimodal imaging. In addition, the high porosity of ZIF–8 makes it possible to load drugs while acting as a contrast agent, thus realizing the integration of diagnosis and treatment. For clinical translation, zinc, as one of the essential trace elements for human body, is considered a highly biocompatible metal ion. Short–term in vivo toxicity experiments also confirmed a promising safety profile [112]. However, the lack of long–term toxicity research remains a key barrier to the clinical application of promising ZIF–8 nanoparticles. Most ZIF–8 nanoparticles can be synthesized easily with high cargo loading through coordination reaction, electrostatic interaction, etc. The pH–sensitive property enables the controlled release of loaded cargo. For other types of nanoparticles such as porous silicon, nanotubes, or superporous hydrogels, various gatekeepers tend to be designed to achieve controlled drug release. However, the inherent pH–responsive property of ZIF–8 enables this process without modification, while avoiding the problem of premature drug leakage. In addition, the toxic effect of ZIF–8 in cancer cells has been reported to be more significant than normal cells [113], which may result from the following two reasons. First, the toxicity of ZIF–8 is mainly derived from the released zinc ions, and cancer cells uptake more zinc ions due to increased permeability. Second, zinc ions have been reported to undergo a Fenton–like reaction with H_2_O_2_, which is highly expressed in cancer cells, thus resulting in the increased production of ROS, and a stronger killing effect on cancer cells.

Current studies on ZIF–8 in cancer applications continue to focus mainly on its combination with phototherapy, and its potential in immunotherapy and GT have attracted increasing attention. Even if it does not carry cargo, ZIF–8 has some ability to improve the immune status of the tumor microenvironment. From the aspect of being a platform for integrated immunotherapy and delivery of gene editing elements, ZIF–8 deserves more exploration in future research. Nowadays, ZIF–8 is often used as a part of the construction of nanosystems. The modifications to the nanoparticles and the consideration of the loaded drugs allow for elaborate nanoparticles to fight cancer from a non–single, systemic novel perspective. A smart nanoplatform that integrates stable controlled release, therapy, and diagnosis is expected to benefit patients in the future. As a kind of MOF material full of potential, ZIF–8 can be expected to be combined with more therapeutic systems in the future and continue to contribute to all aspects of tumor therapy and diagnosis.

## Abbreviations

ZIF	zeolitic imidazolate framework
MOF	metal–organic framework
ST	starvation therapy
GT	gene therapy
MRI	magnetic resonance imaging
CT	computed tomography
PAI	photoacoustic imaging
ROS	reactive oxygen species
PEG	polyethylene glycol
HA	hyaluronic acid
LA	lactobionic acid
FA	folic acid
RGD	Arg–Gly–Asp
PTA	photothermal agent
DOX	doxorubicin
FI	fluorescent imaging
PDA	polydopamine
PTT	photothermal therapy
IR	infrared
NV	nivolumab
CpG	cytosine–phosphate–guanine
ODN	oligodeoxynucleotide
OVA	antigen ovalbumin
GOx	glucose oxidase
HRP	horseradish peroxidase
CHC	α–cyano–4–hydroxycinnamate
PDT	photodynamic therapy
BSA	bovine serum albumin
PAA	polyacrylic acid
PB	Prussian blue
PC	phycocyanin
ICG	indocyanine green
HSP	heat shock protein
RAPA	rapamycin
ICD	immunogenic cell death
IMQ	imiquimod
IR820	new indocyanine green
R837	imiquimod
TAA	tumor–associated antigen
1MT	1–Methyl–D–tryptophan
CO	carbon monoxide
MIT	mitoxantrone
HYD	hydralazine
CDT	chemo–dynamic therapy
